# RNA genome conservation and secondary structure in SARS-CoV-2 and SARS-related viruses: a first look

**DOI:** 10.1261/rna.076141.120

**Published:** 2020-08

**Authors:** Ramya Rangan, Ivan N. Zheludev, Rachel J. Hagey, Edward A. Pham, Hannah K. Wayment-Steele, Jeffrey S. Glenn, Rhiju Das

**Affiliations:** 1 Biophysics Program, Stanford University, Stanford, California 94305, USA; 2 Department of Biochemistry, Stanford University School of Medicine, Stanford, California 94305, USA; 3 Departments of Medicine (Division of Gastroenterology and Hepatology) and Microbiology & Immunology, Stanford School of Medicine, Stanford, California 94305, USA; 4 Department of Chemistry, Stanford University, Stanford, California 94305, USA; 5 Palo Alto Veterans Administration, Palo Alto, California 94304, USA; 6 Department of Physics, Stanford University, Stanford, California 94305, USA

**Keywords:** secondary structure, conservation, SARS-CoV-2, structurome, ncRNA

## Abstract

As the COVID-19 outbreak spreads, there is a growing need for a compilation of conserved RNA genome regions in the SARS-CoV-2 virus along with their structural propensities to guide development of antivirals and diagnostics. Here we present a first look at RNA sequence conservation and structural propensities in the SARS-CoV-2 genome. Using sequence alignments spanning a range of betacoronaviruses, we rank genomic regions by RNA sequence conservation, identifying 79 regions of length at least 15 nt as exactly conserved over SARS-related complete genome sequences available near the beginning of the COVID-19 outbreak. We then confirm the conservation of the majority of these genome regions across 739 SARS-CoV-2 sequences subsequently reported from the COVID-19 outbreak, and we present a curated list of 30 “SARS-related-conserved” regions. We find that known RNA structured elements curated as Rfam families and in prior literature are enriched in these conserved genome regions, and we predict additional conserved, stable secondary structures across the viral genome. We provide 106 “SARS-CoV-2-conserved-structured” regions as potential targets for antivirals that bind to structured RNA. We further provide detailed secondary structure models for the extended 5′ UTR, frameshifting stimulation element, and 3′ UTR. Lastly, we predict regions of the SARS-CoV-2 viral genome that have low propensity for RNA secondary structure and are conserved within SARS-CoV-2 strains. These 59 “SARS-CoV-2-conserved-unstructured” genomic regions may be most easily accessible by hybridization in primer-based diagnostic strategies.

## INTRODUCTION

Severe acute respiratory syndrome coronavirus 2 (SARS-CoV-2) has caused a rapidly expanding global pandemic, with the Coronavirus Disease 2019 (COVID-19) outbreak responsible at the time of writing for over 600,000 cases and 25,000 deaths (Rangan et al. 2020b). The emergence of this pandemic has revealed an urgent need for diagnostic and antiviral strategies targeting SARS-CoV-2. Like other coronaviruses, SARS-CoV-2 is a positive sense RNA virus, with a large RNA genome approaching nearly 30 kilobases in length. Its RNA genome contains protein-coding open reading frames (ORFs) for the viral replication machinery, structural proteins, and accessory proteins. The genome additionally harbors various *cis*-acting RNA elements, with structures in the 5′ and 3′ untranslated region (UTRs) guiding viral replication, RNA synthesis, and viral packaging (Fehr and Perlman 2015). Conserved RNA elements offer compelling targets for diagnostics. In addition, such RNA elements may be useful targets for antivirals, a concept supported by the recent development of antisense oligonucleotide therapeutics and small-molecule RNA-targeting drugs for a variety of targets across infectious and chronic diseases (Spurgers et al. 2008; Connelly et al. 2016; Bennett et al. 2019).

Conserved structured RNA regions have already been shown to play critical functional roles in the life cycles of coronaviruses. Most coronavirus 5′ UTR's harbor at least four stem–loops, with many showing heightened sequence conservation across betacoronaviruses, and various stems demonstrating functional roles in viral replication (Yang and Leibowitz 2015). Furthermore, RNA secondary structure in the 5′ UTR exposes a critical sequence motif, the transcriptional regulatory sequence (TRS), that forms long-range RNA interactions necessary for facilitating the discontinuous transcription characteristic to coronaviruses (van den Born et al. 2005). Beyond the 5′ UTR, the frameshifting stimulation element (FSE) in the first protein-coding ORF (ORF1ab) includes a pseudoknot structure that is necessary for the ratiometric translation of ORF1a and ORF1ab from two overlapping reading frames via programmed ribosomal −1 frame-shifting (Plant and Dinman 2008). In the 3′ UTR, mutually exclusive RNA structures including the 3′ UTR pseudoknot control various stages of the RNA synthesis pathway (Stammler et al. 2011).

**FIGURE 1.**
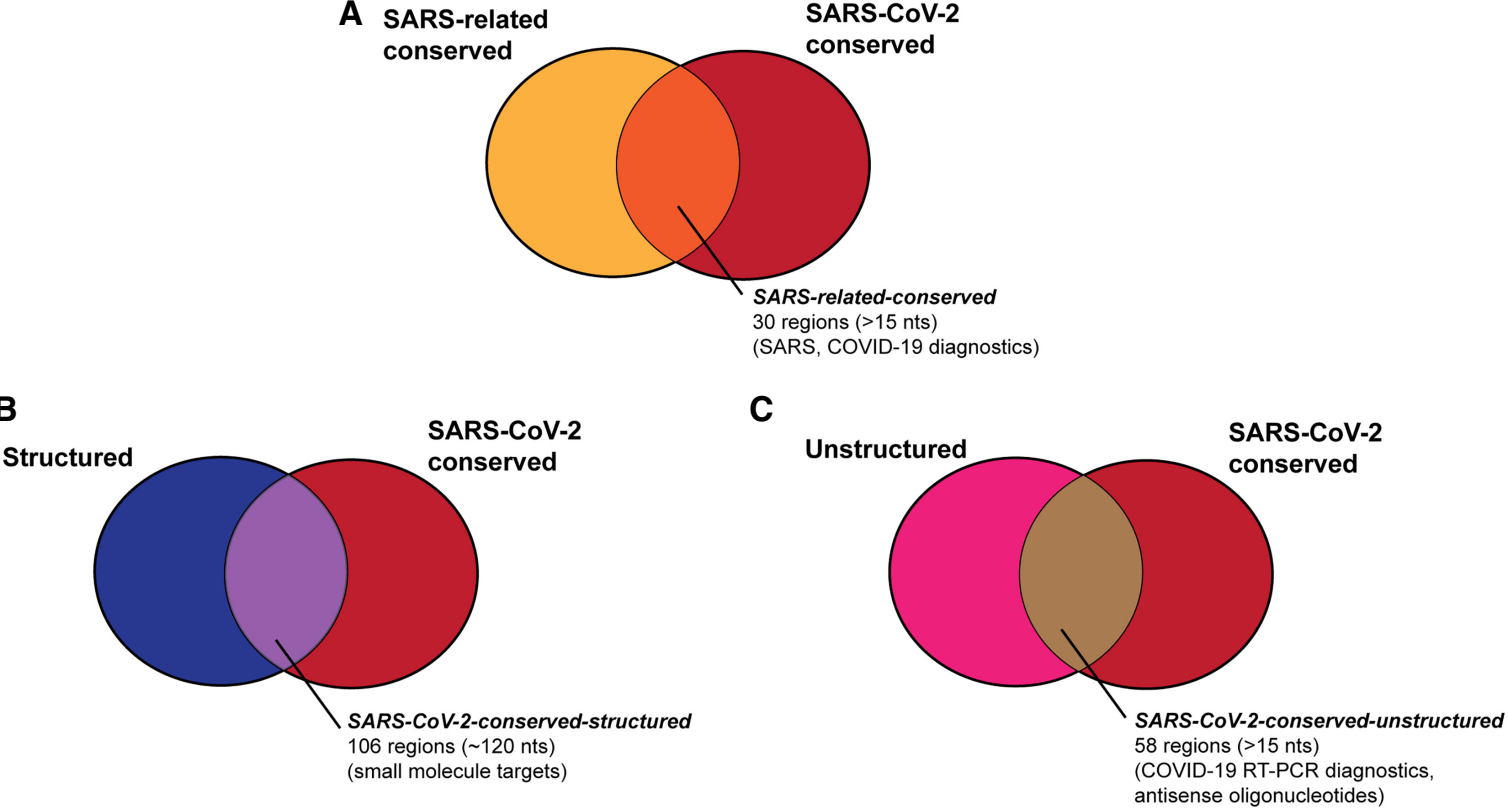
We aim to provide a series of genome regions in SARS-CoV-2 that are useful for a variety of diagnostic and therapeutic strategies, including regions that are (*A*) conserved in SARS-related betacoronaviruses and SARS-CoV-2 sequences (Table 1), (*B*) regions that are structured and conserved in SARS-CoV-2 sequences (Table 2), and (*C*) regions that are unstructured and conserved in SARS-CoV-2 sequences (Table 3).

Beyond these canonical structured regions, the RNA structure of the SARS-CoV-2 genome remains mostly unexplored. Unbiased discovery of other conserved regions and/or structured regions in the virus has the potential to uncover further functional *cis-*acting RNA elements. Here, we analyze RNA sequence conservation across SARS-related betacoronaviruses and currently available SARS-CoV-2 sequences, and we identify structured and unstructured regions that are conserved in each sequence set; these intervals can provide starting points for a variety of diagnostic and antiviral development strategies (Fig. 1). To identify structured regions, we predict maximum expected accuracy structures around conserved regions and report the support of these single structures from predictions of each RNA's structural ensemble. We additionally identify thermodynamically stable secondary structures across the whole genome, finding that currently known structures fall within these predictions, but also identifying new candidate structured regions. We pinpoint unstructured genome intervals by identifying bases with low average base-pairing probabilities. Finally, we present secondary structure models for key RNA structural elements of SARS-CoV-2 annotated in the betacoronavirus family. Many of our conclusions, first reported in the bioRxiv preprint server (Rangan et al. 2020b), have been confirmed in a later bioRxiv report (Andrews et al. 2020), which we discuss briefly.

## RESULTS

### RNA sequence conservation in SARS-related betacoronaviruses and SARS-CoV-2

To identify potential regions of conserved RNA secondary structure in the virus, we located stretches of the SARS-CoV-2 genome with high RNA sequence conservation across SARS-related betacoronavirus full genome sequences. By identifying regions with high RNA sequence conservation as a first step, we reasoned that we would be more likely to filter for functionally relevant structures that must be conserved through virus evolution and thereby discover targets that are potentially less likely to develop resistance against therapeutics or to escape diagnosis as the virus evolves. To ensure reasonable numbers of sequences while still focusing on conservation and structure patterns most relevant to the current pandemic, we chose to analyze not all betacoronaviruses but a subgroup of SARS-related betacoronaviruses. These include SARS, SARS-CoV-2, and SARS-related bat coronaviruses, but not MERS, MHV, or other betacoronaviruses which have been classified into distinct subgroups based on different sequence and structure features in, for example, their 5′ UTR's (Chen and Olsthoorn 2010).

We carried out this analysis beginning with three different sequence alignments. Each captures a range of complete genome sequences across the SARS-related betacoronaviruses, but differ in the total number of sequences and in the redundancy of those sequences, as follows:

1. The first multiple sequence alignment (SARSr-MSA-1) was computed by aligning sequences curated by Ceraolo and Giorgi (2020), filtered by including only the reference genome sequence NC_0405512.2 (Wu et al. 2020) from the SARS-CoV-2 sequence set, removing the two MERS sequences, and leaving in all remaining betacoronavirus whole-genome sequences. This alignment captures a range of SARS-related bat coronavirus and SARS sequences but comprises only 11 sequences. These sequences correspond well to the SARS-related group defined in Gorbalenya et al. (Viruses and Coronaviridae Study Group of the International Committee on Taxonomy of 2020).
2. The second MSA (SARSr-MSA-2) was obtained from BLAST by searching for the 100 complete genome sequences closest to the SARS-CoV-2 reference genome. This alignment captures a larger set of SARS-CoV-2, SARS, and bat coronavirus sequences than SARSr-MSA-1 but includes many sequences with high pairwise similarity.
3. The final MSA (SARSr-MSA-3) was obtained by locating all complete genome betacoronavirus sequences from the NCBI database, and removing mutually similar sequences with a 99% sequence conservation cutoff. With 180 sequences with at most 99% pairwise sequence similarity, this MSA captures a broader set of betacoronaviruses than SARSr-MSA-1 and SARSr-MSA-2 but is more challenging to align due to higher sequence diversity.

We computed conserved regions as contiguous stretches of 15 nt or longer that were 100% conserved (cutoff for SARSr-MSA-1), 98% conserved (cutoff for SARSr-MSA-2), or 54% conserved (cutoff for SARSr-MSA-3). Searching for conserved regions of 15 nt or more enables the design of antisense oligonucleotides that fall within these stretches. The sequence conservation cutoffs chosen ensured that at least 75 candidate conserved stretches were used for further structure analysis for each MSA. When calculating sequence conservation at the 5′ and 3′ sequence ends of the sequence, we did not include sequences that included only leading or trailing sequence deletions up to that point to avoid sequencing artifacts.

**FIGURE 2.**
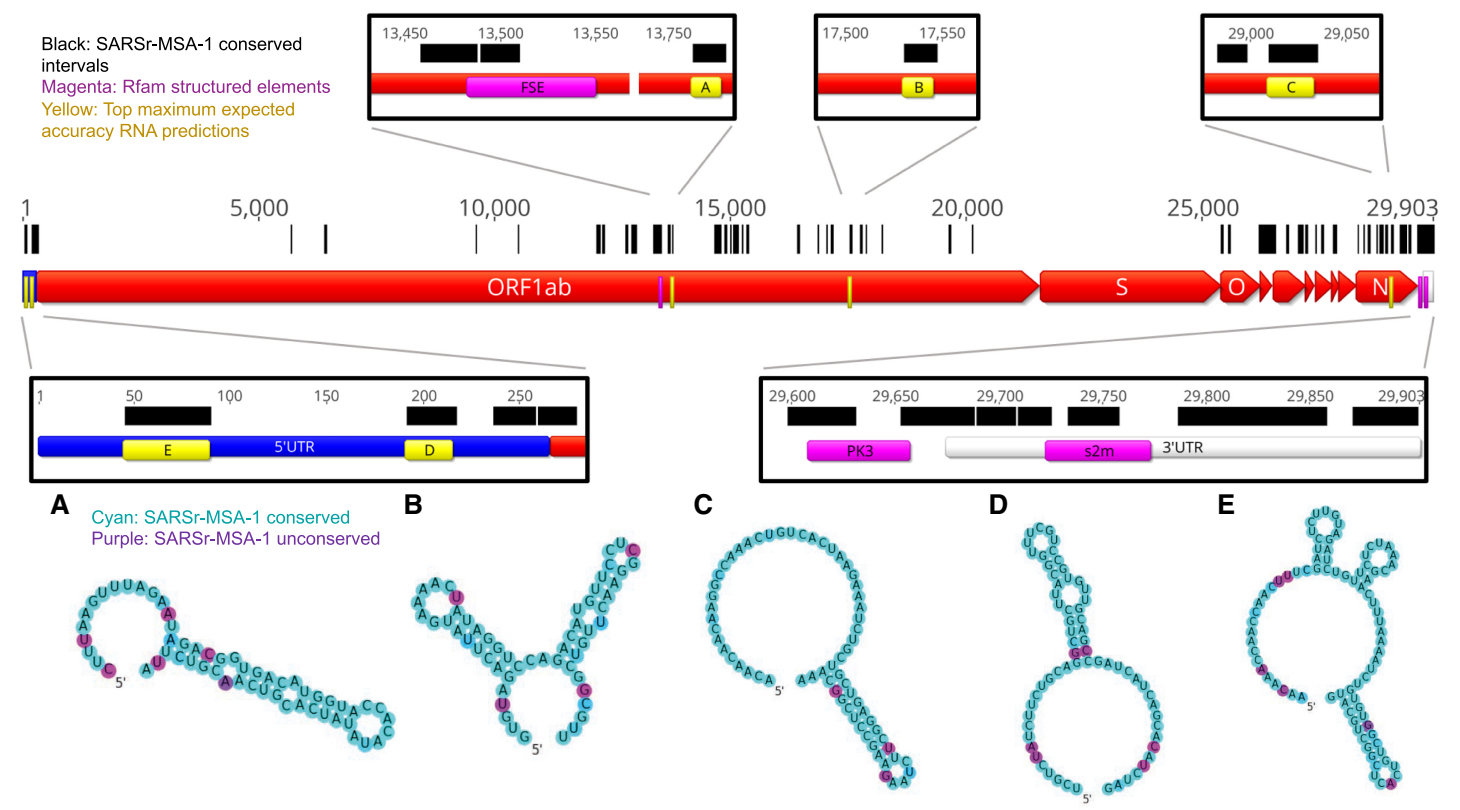
In black we annotate SARSr-MSA-1 conserved regions of the genome, superimposed on SARS-CoV-2 genome ORFs. We depict the top secondary structures as ranked by Matthews correlation coefficient that overlap with these conserved regions, ordered from *A* to *E*. Regions *A* to *E* are annotated on the genome in yellow and are located at genome positions: (*A*) 13743–13798, (*B*) 17511–17566, (*C*) 28990–29054, (*D*) 172–236, (*E*) 26–109. Secondary structures are colored by sequence conservation in SARSr-MSA-1 (cyan = more conserved, purple = less conserved). In magenta are depicted curated Rfam families present in coronaviruses, including the frameshifting stimulation element (FSE), the 3′ UTR pseudoknot (PK3), and the 3′ stem–loop II-like motif (s2m). Figures prepared in Geneious (Kearse et al. 2012) and draw_rna (https://github.com/DasLab/draw_rna).

In Figure 2, we depict conserved regions (100% conservation cutoff, SARSr-MSA-1) alongside the genome coordinates for the reference SARS-CoV-2 sequence. We observe intervals of conservation in the 5′ UTR and 3′ UTR, as expected based on prior work demonstrating sequence conservation surrounding structured RNA elements in these regions (Madhugiri et al. 2014), but we also noted stretches of RNA sequence conservation within some viral ORFs.

Interestingly, in SARSr-MSA-1 and SARSr-MSA-2 we found that conserved stretches overlapped with previously curated Rfam (Kalvari et al. 2018) families for *Coronaviridae* RNA secondary structures: the frameshifting stimulation element (Rfam family RF00507), the 3′ UTR pseudoknot (Rfam family RF00165), and the 3′ stem–loop II-like motif (Rfam family RF00164) (Fig. 2). Locations for the frameshifting stimulation element, 3′ UTR pseudoknot, and 3′ stem–loop II-like motif were confirmed using Infernal (Nawrocki and Eddy 2013), with all regions discovered at an E < 10^−4^ threshold. We also found overlap between conserved stretches and additional 5′ UTR structures that have been established for previous coronaviruses, including the original SARS virus, including stem–loops 2–3 (SL2–3) and stem–loop 5 (SL5) (Yang et al. 2015). These five known RNA structures overlap with conserved regions more than expected; in 10,000 random trials, the chance that five randomly chosen intervals of these lengths all overlap with the conserved regions from SARSr-MSA-1 or SARSr-MSA-2 is less than 0.0003. The enrichment of known RNA structures in these conserved regions suggests that other conserved regions may also harbor RNA structures.

To further tighten this list of conserved sequences to ones most relevant to the current COVID-19 outbreak, we analyzed whether sequence regions conserved across SARS and bat coronaviruses remain conserved in the SARS-CoV-2 strains, most of which emerged after our analysis above (Fig. 1A). We determined the conservation of conserved genome regions from SARSr-MSA-1 across SARS-CoV-2 sequences as of deposition date 03-18-20. For this analysis, we obtained two whole-genome multiple sequence alignments, keeping only full-length genome sequences of at least 29,000 nt in both cases: the first includes 103 NCBI sequences (SARS-CoV-2-MSA-1), and the second includes 739 sequences deposited to GISAID (Elbe and Buckland-Merrett 2017) (SARS-CoV-2-MSA-2). We noted conserved regions in the betacoronavirus alignment SARSr-MSA-1 were more likely to be at least 99% conserved in both SARS-CoV-2-MSA-1 and SARS-CoV-2-MSA-2 than random intervals of the same size (binomial test *P*-value <1 × 10^−5^). Table 1 lists these regions, which we term the SARS-related-conserved regions. These genome regions are conserved across the betacoronavirus sequences in SARSr-MSA-1 and have at least 99% sequence conservation across whole-genome sequences from the SARS-CoV-2 outbreak as of March 18, 2020 (SARS-CoV-2-MSA-2).

**TABLE 1.**
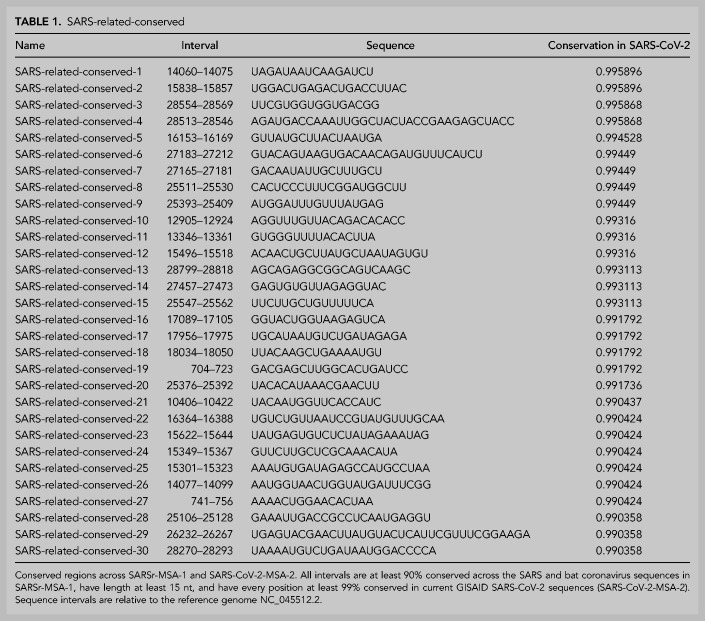
SARS-related-conserved

Conservation percentages for SARSr-MSA-1, SARSr-MSA-2, SARS-CoV-2-MSA-1, and SARS-CoV-2-MSA-2 are included in Supplemental File 1. We expect that some diagnostic and therapeutic strategies will benefit from focusing on conserved regions across a broad range of betacoronaviruses, whereas others may benefit from focusing on regions conserved only in SARS-CoV-2 isolates to date.

### Predictions for structured regions in SARS-CoV-2

The intrinsic RNA structure of a conserved genome region is of interest in current medical research (Fig. 1B). On one hand, stable secondary structure domains are candidates for harboring stereotyped 3D RNA folds that present targets for small-molecule drug therapeutics. On the other hand, if an RNA region is sufficiently unstructured to allow binding by hybridization probes, antisense oligonucleotides may be used to disrupt these functional structures. Such unstructured stretches may also be more likely to be accessible to diagnostic strategies including standard RT-PCR assays (Bustin and Nolan 2004).

We used two approaches to make predictions for conserved structured regions in SARS-CoV-2. First, we predicted RNA structures centered on the most sequence-conserved regions of SARS-related betacoronavirus genomes (alignment SARSr-MSA-1). For each conserved stretch (at least 15 nt long, 100% sequence conservation) along with 20 nt flanking windows, we predicted maximum expected accuracy (MEA) secondary structures using CONTRAfold 2.0 (Do et al. 2006). We then sought to rank sequences based on the predicted probability that the RNA folds into the MEA structure and not other structures. For this ranking, we used the estimated Matthews correlation coefficient (MCC) from each construct's base-pairing probability matrix (Hamada et al. 2010). We note here that while MCC is often used in the RNA structure modeling literature to assess agreement of a prediction with a reference structure, we here use the metric to assess how tightly concentrated the ensemble of predicted secondary structures is to a single predicted secondary structure, the MEA structure. An MEA structure with a higher estimated MCC is expected to have unpaired and paired bases that better align with the construct's predicted ensemble base-pairing probabilities, lending support to the single-structure MEA prediction. In Figure 2 regions A–E, we display the five conserved regions with the top maximum expected accuracy (MEA) secondary structures as ranked by the estimated MCC (all regions listed in Supplemental File 1). Regions D and E occurred within the 5′ UTR and correspond to known SARS-related virus stem–loops SL5a and SL2, respectively. Interestingly, region A is close to but does not overlap with the frameshifting stimulation element; it lies 200 nt downstream from the FSE and could perhaps be involved in a more elaborate structure, as has been described for human coronavirus 229E and other coronaviruses (Herold and Siddell 1993).

We also sought independent methods to identify thermodynamically stable and conserved RNA structures, without initially guiding the search to focus on extremely sequence-conserved genome regions. We made predictions for structured regions using RNAz (Gruber et al. 2010), beginning with the betacoronavirus alignment SARSr-MSA-1. RNAz predicts structured regions that are more thermodynamically stable than expected by comparison to random sequences of the same length and sequence composition (*z*-score), and additionally assesses regions by the support of compensatory and consistent mutations in the sequence alignment (SCI score). These two criteria are combined into a single *P*-score, which when tested empirically on a set of ncRNAs produced a false-positive rate of 4% at a *P* > 0.5 cutoff and 1% at a *P* > 0.9 cutoff. To predict structured regions across the full viral genome, we scanned the SARSr-MSA-1 alignment in windows of length 120 nt sliding by 40 nt, predicted all RNAz hits in the plus strand at a *P* > 0.5 cutoff, clustered the resulting hits to generate maximally contiguous loci of the genome with predicted structure, and filtered results to only include loci with at least one window with a *P* > 0.9 structure prediction.

**FIGURE 3.**
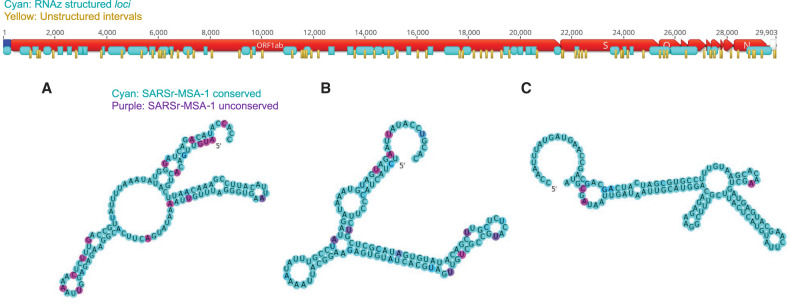
Structured (cyan) and unstructured (yellow) intervals on the genome ORFs for SARS-CoV-2, predicted from RNAz and CONTRAfold 2.0 analysis, respectively. *A*–*C* highlight the three secondary structures for windows that do not overlap with known Rfam or literature-annotated structures with the highest *P*-value scores from RNAz (all *P* > 0.9). These windows are located at genome positions 14207–14366 (*A*), 17126–17245 (*B*), and 26176–26295 (*C*). Secondary structures are colored by sequence conservation (cyan = more conserved, purple = less conserved). Figures prepared in Geneious (Kearse et al. 2012) and draw_rna (https://github.com/DasLab/draw_rna).

The RNAz approach led to the prediction of 44 structured genome loci comprising 117 windows with predicted structure (*P* > 0.9) (Fig. 3). These structured loci cover 46% of the SARS-CoV-2 genome, suggesting that the SARS-CoV-2 genome is highly structured, as has also been found in a recent in silico map of the SARS-CoV-2 RNA structurome (Andrews et al. 2020). We found that five canonical RNA structures (the frameshifting stimulation element, the 3′ UTR pseudoknot, the 3′ UTR hypervariable region, 5′ UTR SL2-3, and 5′ UTR SL5) were present in these loci. Additionally, conserved SARS-CoV-2 regions overlap significantly with predicted RNAz loci, with 62 of 78 SARS-CoV-2 conserved intervals at a 97% sequence cutoff overlapping by at least 15 nt with RNAz loci. This enrichment is statistically significant (*P*-value <0.001 from comparisons to 10,000 random placements of conserved intervals). This enrichment also holds when considering overlaps with conserved regions from SARSr-MSA-1; 124 of the 229 SARSr-MSA-1 conserved intervals at a 90% conservation cutoff overlap by at least 15 nt with RNAz loci (*P*-value 0.0038). This analysis potentially expands the set of conserved structural regions of SARS-CoV-2 beyond known Rfam families and those noted in the literature (full set of RNAz loci in Supplemental File 1). Top-scoring structured windows from RNAz that overlap with conserved sequence regions in SARS-CoV-2-MSA-2 for at least 15 nt are included in Table 2; we termed these SARS-CoV-2-conserved-structured regions. Overlapping intervals between the RNAz predictions and conserved sequence regions in SARSr-MSA-1 are included in Supplemental File 1.

**TABLE 2.**
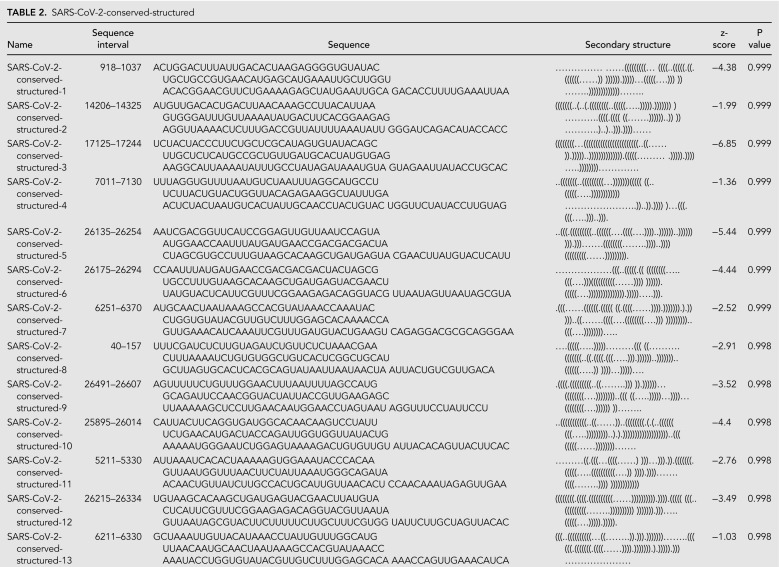

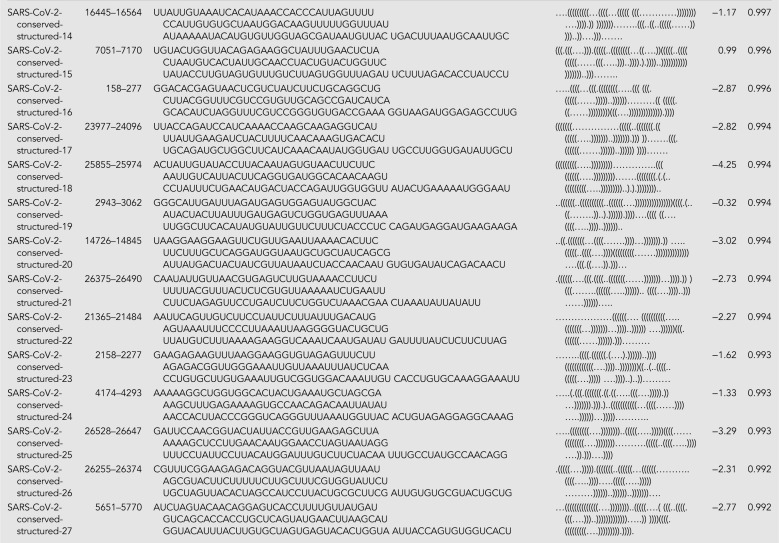

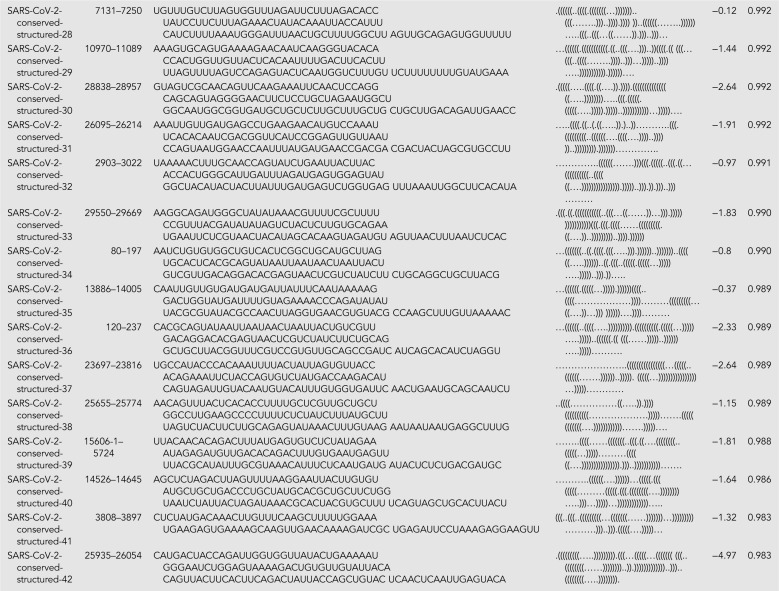

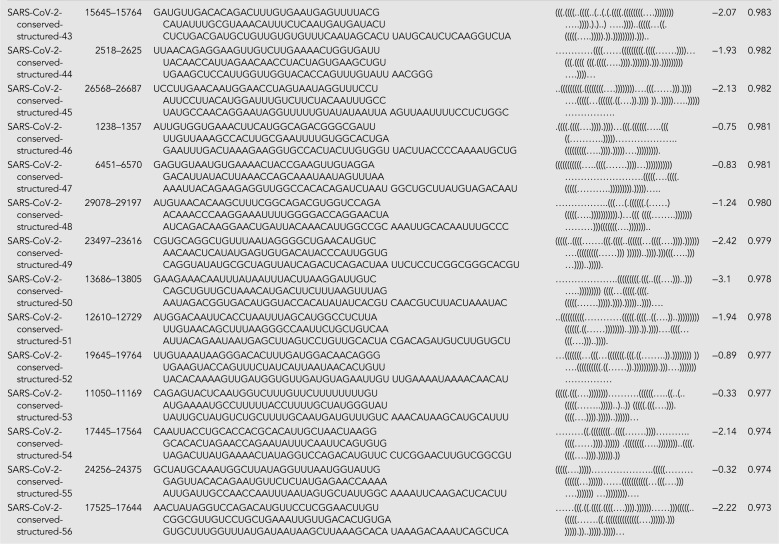

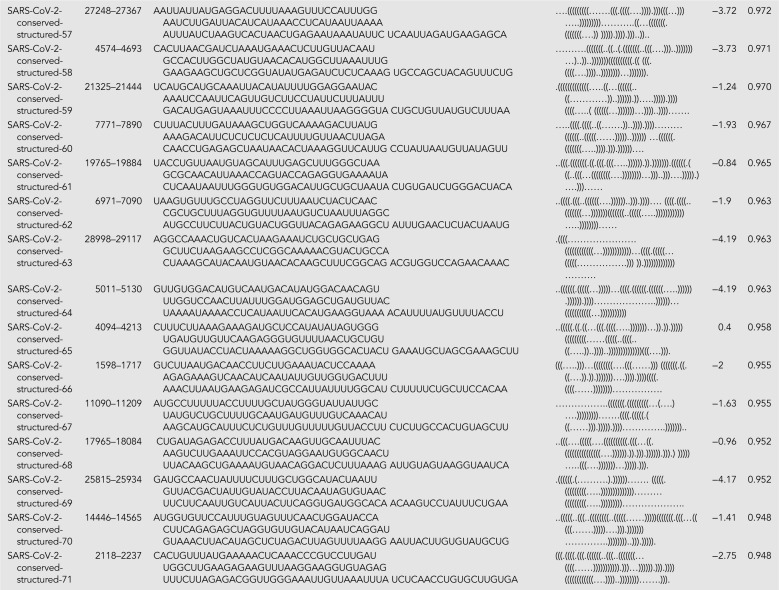

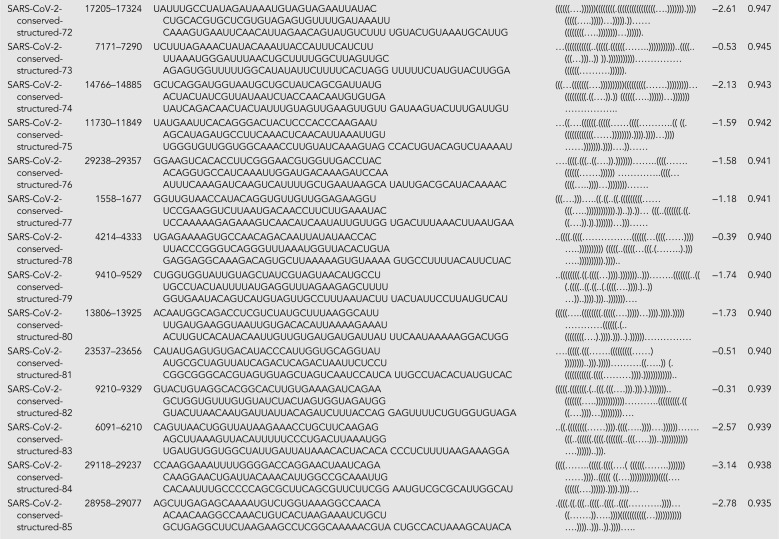

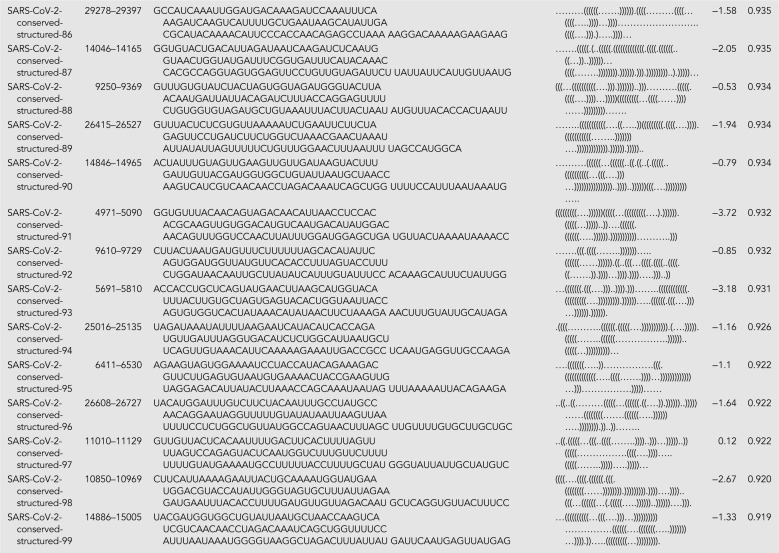

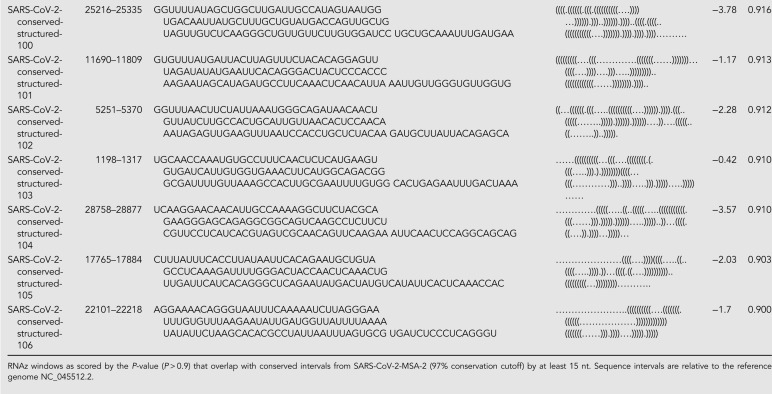
SARS-CoV-2-conserved-structured

We sought to further check structured windows reported by RNAz using orthogonal approaches. First, we explored using R-scape to make structure predictions with covariation signal in the sequence alignments (Rivas et al. 2020). However, we found that the SARSr-MSA-1 alignment had insufficient variation to detect conserved base pairs with covariation, lacking alignment power for all genomic windows (Rivas et al. 2020). Second, we compared windows predicted from RNAz above to those computed from ScanFold in a recent analysis (Andrews et al. 2020). We find that 90 of the 117 RNAz structured windows (*P* > 0.9 cutoff) are predicted ScanFold structured regions that meet a *P*-value threshold of 0.05. This overlap is statistically significant (*P*-value <1 × 10^−5^). Third, we validated structured window predictions with alifoldz, a program that calculates a *z*-score for an alignment window by comparing the window's consensus minimum free energy structure to that of random shuffled alignments. To mirror the RNAz analysis above, we scanned through windows of length 120 nt sliding by 40 nt. We chose a *z*-score cutoff of −2.69, which kept only 1% of windows when running alifoldz on all shuffled windows across the genome. This approach led to predicting 228 alifoldz structured windows, overlapping with 104 of the 117 RNAz structured windows (*P* > 0.9 cutoff). Again, this overlap is statistically significant (*P*-value <1 × 10^−5^). RNAz structured windows supported by alifoldz analysis are highlighted in Supplemental File 1.

#### Conserved unstructured regions of SARS-CoV-2

We additionally located conserved regions of the viral genome predicted to *lack* structure, as such regions may be desired targets for some diagnostic and therapeutic approaches (Fig. 1C). We scanned the SARS-CoV-2 reference genome in windows of length 120 nt sliding by 40 nt, and for each window, we predicted the base-pair probability matrix with CONTRAfold 2.0, using these probabilities to assemble average single-nucleotide base-pairing probabilities across the genome. In Figure 3, we display the 76 stretches of the genome of length at least 15 nt where every base has average base-pairing probability at most 0.4.

It is interesting to note that some structured 120 nt windows reported by RNAz include these unpaired stretches. A simple explanation for this observation is that such regions may encode for well-defined, conserved RNA structures that themselves harbor long unpaired loops to recruit proteins, distal RNA elements, or other molecular machinery.

Overall, we find that 58 of these unpaired stretches have at least 15 nt of overlap with sequence regions that are at least 97% conserved in SARS-CoV-2-MSA-2 (Fig. 5). These unpaired stretches, termed SARS-CoV-2-conserved-unstructured regions, are listed in Table 3 (overlaps with SARSr-MSA-1 are included in Supplemental File 1).

**TABLE 3.**
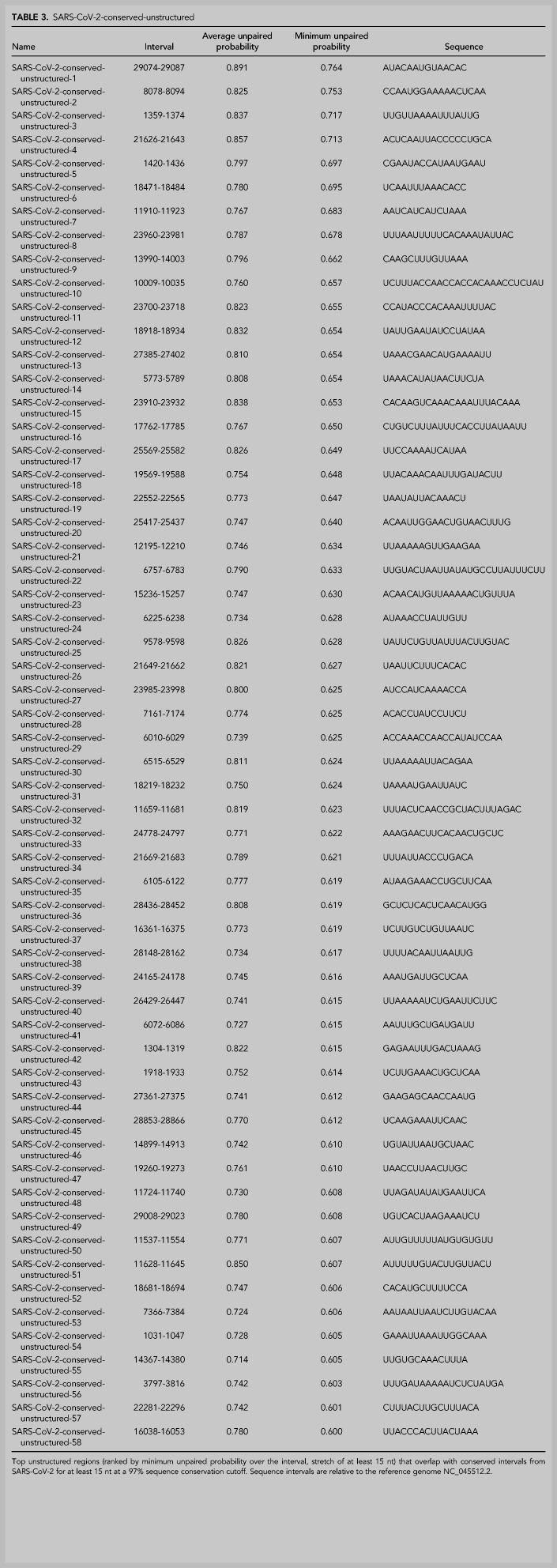
SARS-CoV-2-conserved-unstructured

As an orthogonal check for the unstructured intervals predicted using CONTRAfold 2.0 base-pairing probabilities, we used Vienna's RNAplfold to compute unpaired probabilities for each genome position. In general, we found that RNAplfold predicted lower unpaired probabilities than CONTRAfold 2.0, with only 10 intervals of length at least 15 nt having average base-pairing probability at most 0.4, in contrast with the 76 stretches predicted by CONTRAfold 2.0. Nevertheless, we found that 9 of the 10 intervals predicted by Vienna's RNAplfold overlap with unpaired intervals predicted from our CONTRAfold 2.0 analysis (regions listed in Supplemental File 1).

#### Secondary structure models for canonical structured regions of SARS-CoV-2

**FIGURE 4.**
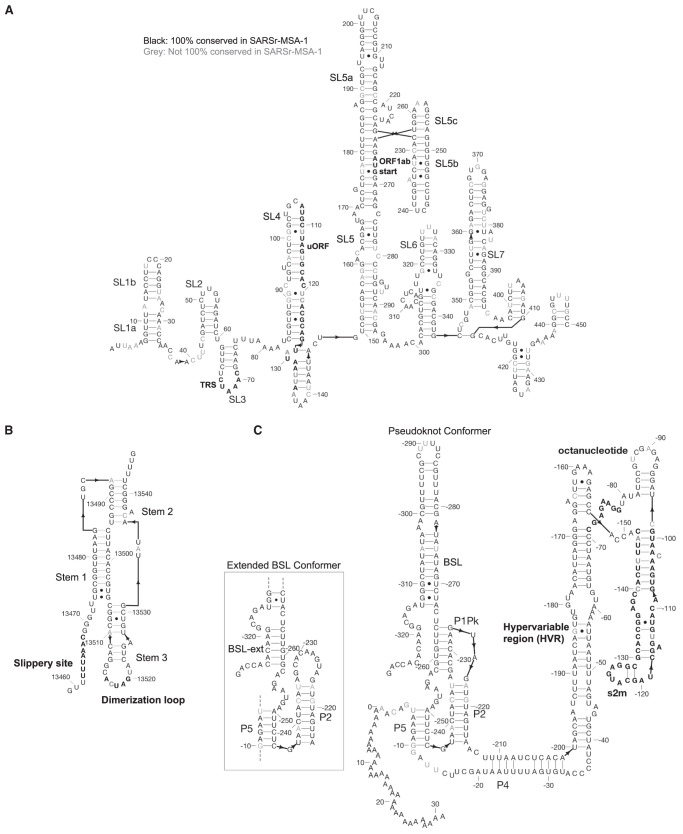
Secondary structure diagrams for (*A*) 5′ UTR, (*B*) frameshifting stimulation element, (*C*) 3′ UTR. Nucleotides are black if 100% conserved in the SARS, bat, and SARS-CoV-2 sequences in SARSr-MSA-1, and gray otherwise. Special labeled domains are in boldface. Structures are based primarily on manual identification of homology with literature coronavirus structure models. Note that numbering in *C* is relative to 3′ end of virus sequence. Figures prepared in RiboDraw (https://github.com/ribokit/RiboDraw).

Currently known RNA structures that recur across betacoronaviruses provide potential starting points for therapeutic development targeting the SARS-CoV-2 RNA genome. Here, we include secondary structures for the 5′ UTR extended partially into the coding region of ORF1ab (Fig. 4A), frameshifting stimulation element (Fig. 4B), and 3′ UTR (Fig. 4C) for SARS-CoV-2. These models are built by analyzing homology with literature-annotated structures in related betacoronaviruses (Chen and Olsthoorn 2010; Wolfinger 2020) and by comparing to predicted secondary structure models from physics-based secondary structure prediction algorithms. The secondary structure for the 5′ UTR is the maximum expected accuracy (MEA) structure predicted by CONTRAfold 2.0. We additionally include computer-readable secondary structures in Table 4 and Supplemental File 1. A brief review of salient secondary structure features in these regions and their putative functional roles in the betacoronavirus life cycle follows.

**TABLE 4.**
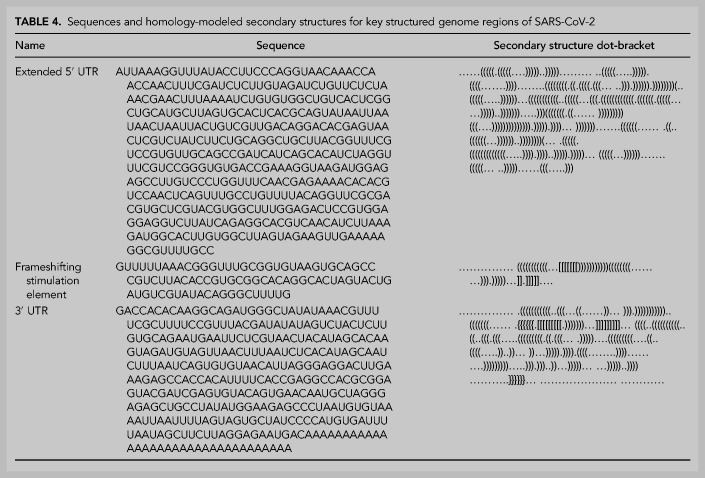
Sequences and homology-modeled secondary structures for key structured genome regions of SARS-CoV-2

The extended 5′ UTR includes five confident stem–loop structures (SL1–SL5), with structures verified by chemical mapping experiments in related coronaviruses (Chen and Olsthoorn 2010; Yang et al. 2015). SL1 and SL2 are conserved across betacoronaviruses, with SL2 having the highest sequence conservation across the 5′ UTR (Madhugiri et al. 2014). The high A-U base-pairing content in the SARS-CoV-2 SL1 sequence and the bulged nucleotides align with prior reports that SL1 is relatively thermodynamically unstable to allow for the formation of long-range interactions (Li et al. 2008). SL2 has been shown to be critical for subgenomic RNA synthesis, with mutations in its conserved pentaloop retaining the production of genome-sized RNA, but not subgenomic RNA segments (Liu et al. 2007). SL3, conserved only in betacoronaviruses, presents the transcription-regulating sequence (TRS) that base pairs with one of several complementary sequences in nascent negative-sense strands in a “copy-choice mechanism” that gives rise to discontinuous transcription of subgenomic mRNAs (van den Born et al. 2005). SL4 contains a short upstream ORF, here labeled uORF, which precedes the first longer ORF1ab of the genome. The uORF leads to attenuated transcription of ORF1ab that appears helpful but is not essential for viral replication (Madhugiri et al. 2014). SL5 has been implicated in a potential role in viral packaging, and contains the AUG start codon for long ORF1ab which encodes the viral replicase/transcriptase polyprotein. The SARS-CoV-2 SL5 domain has common features with the domain in other group IIb betacoronaviruses, for instance including UUCGU pentaloops on SL5a and SL5b, and a GNRA tetraloop on SL5c (Chen and Olsthoorn 2010). Prior DMS-probing data for Stem 5 in SARS-CoV aligned with the proposed SL5a,b,c structures (Chen and Olsthoorn 2010). Two additional stems (SL6 and SL7) are predicted in the MEA structure from CONTRAfold 2.0, but prior literature has not established whether such stems embedded in the coding region are functionally important across betacoronaviruses.

The frameshifting stimulation element (FSE) is located in ORF1ab and is involved in regulating a (−1) ribosomal frameshift event that is necessary for completing translation of ORF1ab. The FSE consists of a conserved pseudoknot structure that regulates the rate of ribosomal frameshifting at an upstream slippery site (Plant and Dinman 2008). This domain is nearly exactly conserved between SARS-CoV and SARS-CoV-2, suggesting a similar mechanism for ribosomal pausing and slippage between the two viruses (Kelly and Dinman 2020).

The 3′ UTR contains various domains critical for regulating viral RNA synthesis and potentially translation. The most 5′ region of the 3′ UTR includes a switch-like domain involving mutually exclusive formation of a pseudoknot and stem–loop, both of which are essential for viral replication with putative roles in establishing the kinetics of RNA synthesis (Goebel et al. 2004; van den Born et al. 2005). The hyper-variable region (HVR) is not essential for viral RNA synthesis, as this can be removed while allowing for viral replication in tissue culture; however, viruses without this domain have lower pathogenicity in mice (Goebel et al. 2007). This domain contains a completely conserved octonucleotide sequence with unconfirmed functional significance. The stem–loop II-like motif (s2m) is another subregion of the HVR that is conserved in SARS-CoV-2 and other coronaviruses. A crystal structure of the SARS s2m domain has been shown to be homologous to an rRNA loop that binds translation initiation proteins, leading this domain to have a proposed role in recruiting host translational machinery (Robertson et al. 2005). The domain has been proposed to be a selfish element due to its recurrence in numerous virus families outside the *Coronaviridae*, but its function is not well understood (Jonassen et al. 1998).

### DISCUSSION

**FIGURE 5.**
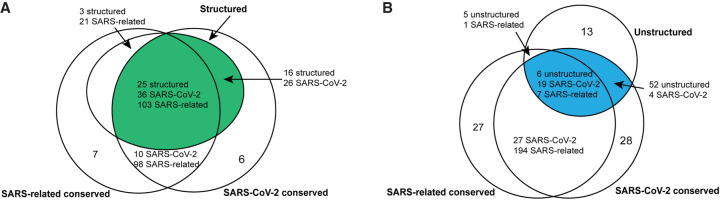
We depict the predicted number of structured, unstructured, and conserved intervals for a choice of sequence conservation cutoffs. The SARS-related conserved intervals are all regions of at least 15 nt with each position at least 90% conserved across an alignment of SARS, bat coronavirus, and SARS-CoV-2 sequences (SARSr-MSA-1). The SARS-CoV-2 intervals are regions of at least 15 nt with each position at least 97% conserved across an alignment of currently available SARS-CoV-2 sequences (SARS-CoV-2-MSA-2). Structured intervals are loci predicted from RNAz with some loci containing multiple RNAz windows, and unstructured intervals are stretches of at least 15 nt where all bases have base-pairing probability at most 0.4. All interval intersections are required to have at least 15 nt overlaps, with the number of overlapping intervals listed for each interval type involved in the intersection. Top-scoring structured intervals conserved in SARS-CoV-2 sequences (green) are listed in Table 2. Top-scoring unstructured intervals conserved in SARS-CoV-2 sequences (blue) are listed in Table 3.

Understanding the RNA structure of the SARS-CoV-2 genome can guide RNA-targeting interventions and diagnostic development. Here we have presented an initial analysis of RNA sequence conservation across betacoronaviruses and current SARS-CoV-2 sequences, predictions for structured and unstructured domains of the viral RNA genome, and homology-derived secondary structure models for previously established coronavirus structured elements that recur in the SARS-CoV-2 genome: the extended 5′ UTR, the frameshifting stimulation element, and the 3′ UTR. By filtering for sequences that have more than one of these properties, we have curated three sets of RNA genomic regions of potential interest for further structural analysis, which we have termed the SARS-related-conserved, SARS-CoV-2-conserved-structured, and SARS-CoV-2-conserved-unstructured sets. Figure 5 gives a more extensive presentation of how these sets overlap. Our hope is that these steps will provide useful starting points for efforts to develop antivirals and diagnostics that depend on targeting either structured or unstructured viral genomic regions.

The alignments used here act as an initial starting point for evaluating RNA sequence conservation in SARS-related viruses and in SARS-CoV-2 strains. Further conservation analysis may benefit from exploring the use of codon-correct viral genome alignments (Libin et al. 2019) or reference-guided alignments (Tzou et al. 2017). Additionally, alignments capturing a broader range of betacoronavirus sequences may enable the use of covariation signals for secondary structure prediction with tools like R-scape (Rivas et al. 2017).

The abundant RNA structures involved in the replication cycle of betacoronaviruses present ample opportunities for therapeutic development, but our analysis is not complete. First, while homology with prior structures annotated in betacoronaviruses lends some confidence to the 5′ UTR, frameshifting stimulation element, and 3′ UTR secondary structures presented here, there may still be some inaccuracies. For instance, SL6 and SL7 in the 5′ UTR are built based on computer modeling. As another example, the frameshifting stimulation element structure presented here differs in two base pairs compared to that presented by Kelly and Dinman (2020). While a recent analysis of the SARS-CoV-2 structurome conducted with ScanFold (Andrews et al. 2018) supports various base pairs in the homology models for the 5′ UTR, FSE, and 3′ UTR presented here, base pairs in the SL5 basal stem of the 5′ UTR and in the hyper-variable region of the 3′ UTR are not predicted by ScanFold (Andrews et al. 2020). Some of these base pairs would not be found by ScanFold because they involve pairing of nucleotides that extend beyond the ScanFold window size used (120 nt), as is the case for the SL5 basal stem in the extended 5′ UTR and the basal portion of the 3′ UTR hyper-variable region stem. Some other base pairs involved in pseudoknots would not be found by ScanFold. However, numerous base pairs in the hyper-variable region model presented here (29695–29809 in numbering based on reference genome NC_0405512.2 [Wu et al. 2020], or −117 to −63 in numbering corresponding to Fig. 4C) that could be identified by ScanFold windowed analysis were not found by ScanFold; it is possible that RNA in these regions adopts a heterogeneous set of secondary structures or a tertiary structure stabilized by noncanonical pairs that are not modeled in ScanFold. Additional biochemical and genetic verification, particularly through compensatory mutagenesis, will be needed to further assess these structures.

Beyond the secondary structures highlighted here, prior work has pinpointed a variety of RNA–RNA interactions important to the betacoronavirus life cycle. Long-range interactions between the 5′ UTR and 3′ UTR have been implicated in RNA synthesis, for instance with mutations in the 5′ UTR SL1 only supporting viral replication when coevolving with specific mutations in the 3′ UTR (Guan et al. 2012; Madhugiri et al. 2014). Such long-range RNA–RNA interactions would be missed in our analyses above which have focused on shorter windows. In related coronaviruses, RNA structures that act as packaging signals have been identified in genomic ORFs (Madhugiri et al. 2014). Such packaging signals may reside in regions identified here from our RNAz computational analysis or may have been missed. Last, it seems likely that the reverse complement of the RNA genome harbors important functional elements which we have not analyzed in detail in this study.

We believe a more thorough structure/function analysis of the virus should be obtainable with recent experimental technologies that integrate multidimensional chemical mapping, electron microscopy, and computer modeling (Kappel et al. 2019; Zhang et al. 2019). New candidates for structured RNA elements in SARS-CoV-2 could play various functional roles, perhaps regulating viral packaging, replication, RNA synthesis, or translation initiation. To further improve these structure predictions, information about protein-binding events should be integrated, and biochemical assays can be conducted with these proteins present or even in cells. Accounting for protein-binding events will be critical to completing a picture of accessible and structured RNA sites.

The secondary structures predicted here present starting points for 3D modeling of RNA-only structures in various regions, including the 5′ UTR SL5, the frameshifting stimulation element, the 3′ UTR pseudoknot, and the 3′ stem–loop II-like motif, and this modeling has begun (Rangan et al. 2020a). Furthermore, these regions and novel predicted structures can serve as candidates for RNA-only structure determination. Such 3D structures have the potential to reveal well-defined 3D folds with conserved binding domains for small-molecule drugs, potentially presenting alternative approaches for targeting SARS-CoV-2.

### MATERIALS AND METHODS

#### Open reading frame (ORF) annotation

The ORFs included in Geneious (Kearse et al. 2012) genome maps (Figs. 2, 3) were obtained from protein annotations deposited in NCBI for the reference genome NC_0405512.2 (Wu et al. 2020). We exclude ORF10, as recent reports have found no evidence for ORF10 expression in SARS-CoV-2 (Kim et al. 2020).

#### Conservation analysis

Three alignments for SARS-related viruses were prepared:

1. SARSr-MSA-1 was generated by realigning the sequences curated by Ceraolo and Giorgi (Ceraolo and Giorgi 2020) with MUSCLE (Edgar 2004) using default alignment settings, excluding all nonreference genome copies of the SARS-CoV-2 sequence and excluding MERS sequences JX869059.2 and KT368829.1.
2. SARSr-MSA-2 was generated by downloading the MSA provided by BLAST for the top 100 complete genome sequences closest to the SARS-CoV-2 reference genome.
3. SARSr-MSA-3 was generated by obtaining all complete betacoronavirus genome sequences available from the NCBI database, removing mutually similar sequences using a 99% cutoff with CD-HIT-EST (Li et al. 2001), and computing an MSA with Clustal Omega (Sievers et al. 2011) using default settings.

Two alignments for SARS-CoV-2 sequences were prepared:

1. SARS-CoV-2-MSA-1 was generated by downloading the MSA provided by NCBI for the 103 whole-genome SARS-CoV-2 sequences deposited as of 03-18-20.
2. SARS-CoV-2-MSA-2 was generated from 739 GISAID (Elbe and Buckland-Merrett 2017) sequences, including all sequences described in the Nextstrain project metadata file as of 03-18-20 (https://github.com/nextstrain/ncov). Sequences were aligned using MAFFT (Katoh and Frith 2012) with the –add flag to add GISAID sequences to seed alignment SARS-CoV-2-MSA-1, and the sequences in SARS-CoV-2-MSA-1 were subsequently removed to avoid duplicates.

All alignments are included in the associated GitHub repository: https://github.com/DasLab/SARSCoV2_Secstruct_Cons.

#### Analysis of structured elements

To identify regions in the SARS-CoV-2 reference genome NC_0405512.2 (Wu et al. 2020) that were matches to Rfam (Kalvari et al. 2018) families, we used Infernal (Nawrocki and Eddy 2013) to build covariance models from Rfam families RR0164, RR0165, and RR0507 with cmbuild, and we ran cmscan to find hits with an E < 1 × 10^−4^ threshold.

MEA structures were computed using CONTRAfold 2.0 (Do et al. 2006) for 20 nt flanking windows around conserved intervals in SARS-CoV-2. To estimate the Matthews correlation coefficient of these single-structure predictions, we computed the pseudo-expected Matthews correlation coefficient as previously described (Hamada et al. 2010). Base-pairing probability matrices were computed with CONTRAfold 2.0, and these were then used to calculate the expected number of true positive, true negative, false positive, and false negative base pairs. These computations were carried out using the Arnie package (https://github.com/DasLab/arnie).

RNAz (Gruber et al. 2010) structures were predicted in windows of the SARS-CoV-2 genome using the SARSr-MSA-1 alignment. We used rnazWindow.pl to compile alignment windows across SARSr-MSA-1 with at least four sequences in each window, using a window size of 120 nt sliding by 40 nt, and using default settings otherwise. RNAz hits were computed at the *P* > 0.5 threshold for the forward strand with z-scores computed without a shuffled sequence background for efficiency, using the –no-shuffle flag. The resulting RNAz structured windows were then clustered with rnazCluster.pl, filtered with rnazFilter.pl at a *P* > 0.9 threshold, and sorted with rnazSort.pl.

We ran alifoldz (Washietl and Hofacker 2004) on the same genome windows used with RNAz above, again using SARSr-MSA-1. The alifoldz z-score computations were calculated for the forward strand only with alifoldz.pl. We additionally calculated alifoldz z-scores for alignment windows that were shuffled with shuffle-aln.pl to assess background z-scores, determining that 1% of shuffled alignment *z*-scores were less than −2.69.

We computed alignment powers with R-scape (Rivas et al. 2017) to assess the potential for using SARSr-MSA-1 for covariation analysis. We generated Stockholm alignment files with biopython (Cock et al. 2009) for windows of 120 nt each sliding by 40 nt. For each window, we ran R-scape with the –fold flag to predict new structures, obtaining estimates for the power of each base pair in the predicted structure (here, power is the expected sensitivity for detecting base pairs given the number of substitutions in the alignment at that base pair). We then averaged across base pairs in each structure to obtain the alignment power as described previously (Rivas et al. 2020), noting that all windows’ alignment powers fell below the 0.10 threshold used to distinguish low-power from high-power alignments.

#### Analysis of unstructured elements

To obtain probabilities that each genome position in SARS-CoV-2 was unpaired, we computed base-pairing probability matrices with CONTRAfold 2.0 (Do et al. 2006) in windows of 120 nt sliding by 40 nt, and for each genome position, we summed the probabilities of pairing with all potential partners. We then averaged these nucleotide pairing probabilities across all windows that nucleotide was present in. Additionally, RNAplfold (Bernhart et al. 2006) was run with window size 120 nt, producing another set of unpaired probabilities for each position in the genome.

### DATA DEPOSITION

Code used for the conservation, structured, and unstructured analyses above can be found at the GitHub repository: https://github.com/DasLab/SARSCoV2_Secstruct_Cons. The repository additionally includes alignment files, Rfam families and covariance models, and output from the RNAz, R-scape, alifoldz and RNAplfold analyses.

### SUPPLEMENTAL MATERIAL

Supplemental material is available for this article.

## Supplementary Material

Supplemental Material
