# Supplementary material for: RNA genome conservation and secondary structure in SARS-CoV-2 and SARS-related viruses: a first look
Source: RNA. 2020 Aug;26(8):937–59. doi: 10.1261/rna.076141.120 (PMC7373990; doi:10.1261/rna.076141.120)
Supplement: Supplemental Material [file supp_26_8_937__index.html]

RNA genome conservation and secondary structure in SARS-CoV-2 and SARS-related viruses: a first look — RNA genome conservation and secondary structure in SARS-CoV-2 and SARS-related viruses: a first look — Supplemental Material 

# RNA genome conservation and secondary structure in SARS-CoV-2 and SARS-related viruses: a first look

## Supplemental Material

- Supplemental\_File1.xlsx
